# Sympathetic regulation of vascular function in health and disease

**DOI:** 10.3389/fphys.2012.00284

**Published:** 2012-07-24

**Authors:** Rosa M. Bruno, Lorenzo Ghiadoni, Gino Seravalle, Raffaella Dell'Oro, Stefano Taddei, Guido Grassi

**Affiliations:** ^1^Department of Internal MedicineUniversity of Pisa, Italy; ^2^Institute of Clinical Physiology - CNRPisa, Italy; ^3^Istituto Auxologico ItalianoMilan, Italy; ^4^Clinica Medica, Dipartimento di Medicina Clinica, Prevenzione e Biotecnologie Sanitarie, Università Milano-Bicocca, Ospedale San GerardoMonza, Milan, Italy; ^5^Istituto a Carattere Scientifico IRCCS Multimedica, Sesto San GiovanniMilan, Italy

**Keywords:** microneurography, vascular function, endothelium, arterial stiffness, nitric oxide

## Abstract

The sympathetic nervous system (SNS) is known to play a pivotal role in short- and long-term regulation of different functions of the cardiovascular system. In the past decades increasing evidence demonstrated that sympathetic neural control is involved not only in the vasomotor control of small resistance arteries but also in modulation of large artery function. Sympathetic activity and vascular function, both of which are key factors in the development and prognosis of cardiovascular events and disease, are linked at several levels. Evidence from experimental studies indicates that the SNS is critically influenced, at the central and also at the peripheral level, by the most relevant factors regulating vascular function, such as nitric oxide (NO), reactive oxygen species (ROS), endothelin (ET), the renin-angiotensin system. Additionally, there is indirect evidence of a reciprocal relationship between endothelial function and activity of the SNS. A number of cardiovascular risk factors and diseases are characterized both by increased sympathetic outflow and decreased endothelial function. In healthy subjects, muscle sympathetic nerve activity (MSNA) appears to be related to surrogate markers of endothelial function, and an acute increase in sympathetic activity has been associated with a decrease in endothelial function in healthy subjects. However, direct evidence of a cause-effect relationship from human studies is scanty. In humans large artery stiffness has been associated with increased sympathetic discharge, both in healthy subjects and in renal transplant recipients. Peripheral sympathetic discharge is also able to modulate wave reflection. On the other hand, large artery stiffness can interfere with autonomic regulation by impairing carotid baroreflex sensitivity.

## Introduction

The sympathetic nervous system (SNS), one of the two divisions of the autonomic nervous system, is known to play a central role in cardiovascular homeostasis (Wallin and Charkoudian, 2007). In particular, SNS is the effector of neurogenic control of vascular tone, inducing mainly vasoconstriction of small resistance arteries. It is well established that SNS is mainly involved in short-term regulation of vasomotor tone and blood pressure (BP), allowing fast adaptation to different physiological conditions by means of the classical autonomic reflexes, in order to maintain cardiovascular homeostasis (Wallin and Charkoudian, 2007). Increasing evidence suggests that sympathetic activity also plays a key role in long-term BP control (Joyner et al., 2008; Fink, 2009). Experimental studies demonstrated that sympathetic activation may induce sustained BP increases by several mechanisms (Joyner et al., 2008; Fink, 2009; Grassi, 2009). In the last few years, novel non-pharmacological antihypertensive approaches targeting SNS have been developed in humans, including renal denervation and baroreceptor-activating therapy, highlighting the clinical relevance of autonomic modulation of BP (Unger et al., 2011).

Recent indirect and direct evidence suggests that sympathetic activity and vascular function, which are both key factors in the development and prognosis of cardiovascular events and disease, could be linked in a more complex fashion. In particular, the same pathways are involved in central and peripheral autonomic regulation as well as in vascular function regulation, suggesting that vascular homeostasis is maintained through pathways activated by the same signaling both at the level of the autonomic nervous system and in the vascular milieu, allowing an integrated, multidistrict response (Grassi, 2001; Patel et al., 2001; Bruno et al., 2011; Hirooka et al., 2011). Furthermore, SNS may directly modulate functional and mechanical properties of large arteries. This is suggested by the evidence that markers of vascular function are inversely related to various measures of sympathetic discharge (Sverrisdottir et al., 2010; Swierblewska et al., 2010), and it is in line with the induction of endothelial dysfunction by sympatho-excitatory maneuvers (Padilla et al., 2010). Adrenergic activation is also chronically present in several cardiovascular diseases: this represents a detrimental and maladaptive phenomenon, possibly inducing chronic changes in vascular function and structure, namely vascular remodeling (Grassi et al., 2009). Moreover, chronic adrenergic hyperactivity is involved in the pathogenesis of several cardiovascular risk factors, thus indirectly inducing vascular dysfunction and damage (Lembo et al., 1992; Joyner et al., 2008). Sympathetic activity may induce sustained increases in BP through several mechanisms, e.g., by causing peripheral vasoconstriction, potentiating cardiac contraction, reducing venous capacitance, affecting renal sodium, and water excretion, through baroreflex dysfunction (Fink, 2009). A higher sympathetic tone could also favor weight gain, hyperinsulinemia and altered glucose metabolism (Lembo et al., 1992; Joyner et al., 2008), all of which are conditions heavily compromising vascular function and structure. Microneurography is a technique allowing direct recording of sympathetic efferent post-ganglionic discharge directed to various different districts (Grassi and Esler, 1999). In particular it is used to record from a peripheral nerve the sympathetic discharge controlling vasomotor tone in muscle vascular districts in humans, the so-called muscle sympathetic nerve activity (MSNA) (Grassi and Esler, 1999). Though limited by technical difficulty and by invasiveness, microneurography has many advantages: it is highly reproducible over several years, it is closely related to sympathetic traffic directed to other districts such as the brain, heart, and kidney, it can be repeated over time to assess effects of interventions, it allows direct quantification of sympathetic nerve traffic regulating vasomotor tone and study of instantaneous reactions to rapid stimuli (Vallbo et al., 2004). For these reasons, it is an irreplaceable tool in order to comprehend physiological mechanisms underlying autonomic reflexes and to reveal relationships between peripheral neural activity and various functions, including vascular function.

This review is aimed at evaluating the state-of-the art on the sympathetic regulation of vascular function in health and disease. In particular, the relationship with endothelial dysfunction, which is an alteration specifically due to reduced nitric oxide (NO) bioavailability in endothelial cells, and arterial stiffness, a process with both a functional and structural basis occurring mainly in large arteries and critically influenced by endothelium-derived substances, is explored. Evidence derived from experimental animal studies, including microneurographic studies, is reviewed for the section regarding common regulating pathways between SNS and vascular function. The subsequent sections are mainly focused on human studies performed by means of microneurography, although studies performed with different techniques are also quoted whenever appropriate.

## SNS and vascular function: common regulating pathways

**Figure 1:**
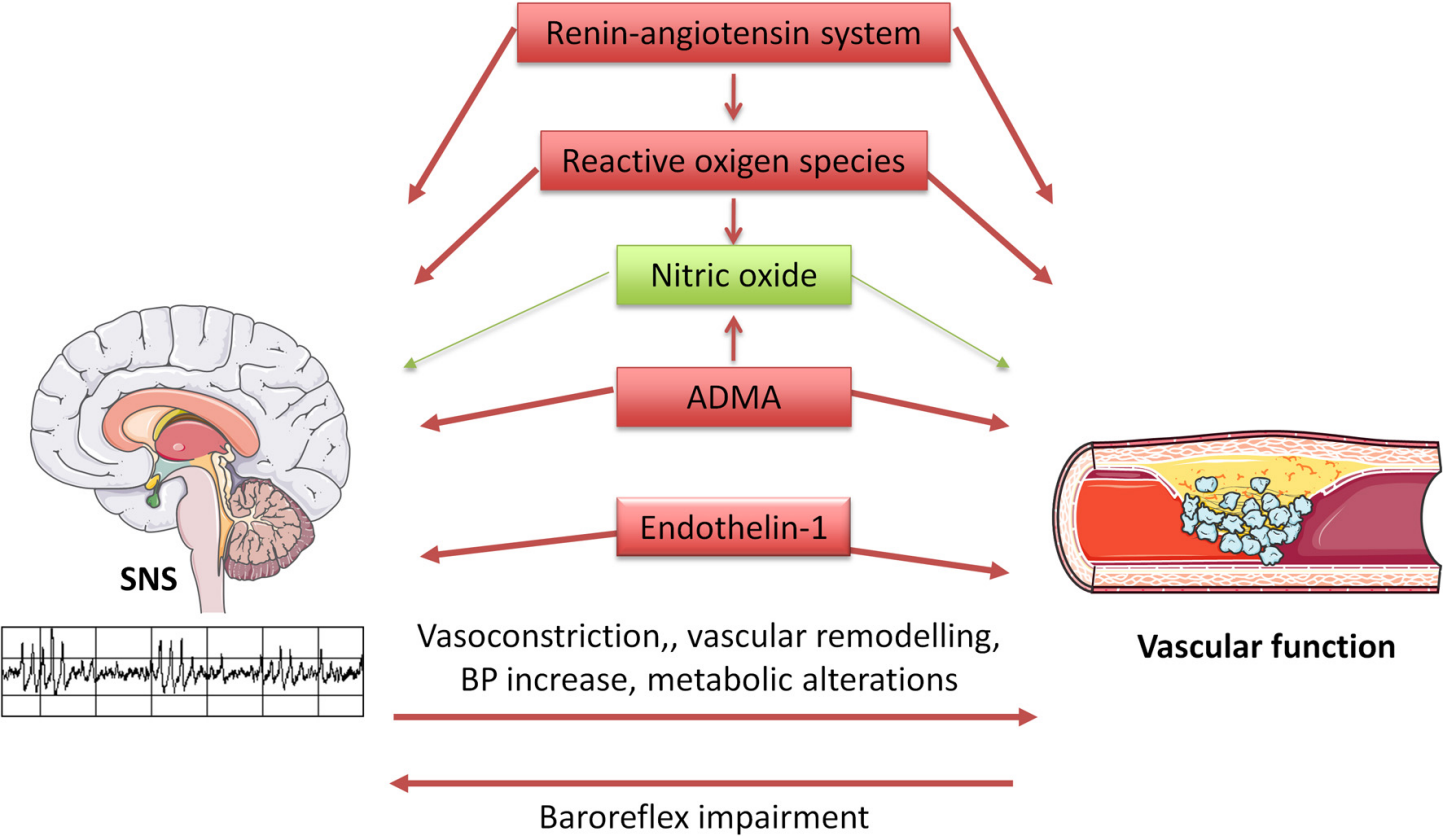
**Diagram illustrating interrelationships between the sympathetic nervous system (SNS) and vascular function.** SNS influences vascular function through multiple mechanisms, including direct vasoconstriction and wall remodeling, blood pressure increase, and metabolic alterations. In turn, arterial stiffness seems to induce baroreflex impairment, thus altering SNS regulation. Furthermore, the same regulatory systems are involved in vascular and SNS homeostasis, with beneficial (in green) and detrimental (in red) effects.

Evidence from experimental studies indicates that the SNS is critically influenced, both at the central and peripheral level, by the most relevant factors regulating vascular function: NO, reactive oxygen species (ROS), endothelin (ET), the renin-angiotensin system (Figure 1).

### Nitric oxide

NO is probably the most important molecule produced by the endothelium. NO is produced from the aminoacid L-arginine by an enzyme known as NO synthase (NOS), which is present in three isoforms in different tissues (Luscher and Vanhoutte, 1990; Bruno and Taddei, 2011). Neuronal NOS (nNOS) is constitutively expressed in neurons both in the central and in the peripheral nervous system: NO is in effect an important neurotransmitter cooperating in autonomic regulation of cardiovascular function (Patel et al., 2001; Hirooka et al., 2011). nNOS is also present in macrophages and endothelial cells, where it seems to play a role in regulation of basal vascular tone (Seddon et al., 2008). Inducible NOS (iNOS) is expressed in a number of different cellular types: its activity is negligible in baseline conditions, but it is steadily induced by inflammatory stimuli (Luscher and Vanhoutte, 1990). Endothelial NOS (eNOS) is a constitutive enzyme isoform, first discovered in endothelial cells, but also found in neuronal cells. NO release from endothelium is determined by receptor-mediated mechanisms (acetylcholine, bradikynin, serotonin, substance P, adenosine diphosphate), but also by mechanical stimuli. In particular shear stress, namely tangential cyclic stress generated on vascular walls by blood flow, is the most powerful mechanism of stimulated NO release (Bruno and Taddei, 2011). The main stimuli negatively influencing eNOS expression are hypoxia, tumor necrosis factor-α, inflammatory cytokines (Luscher and Vanhoutte, 1990). eNOS can be also inhibited by false substrates such as N-monomethyl-L-arginine (L-NMMA), which are commonly used to test the degree of endothelium-dependent vasodilation. Asymmetric dimethylarginine (ADMA), a naturally occurring aminoacid, is an endogenous inhibitor of eNOS, which can cause endothelial dysfunction and is associated with increased cardiovascular risk (Bruno and Taddei, 2011).

It is now well established that NO acts as a sympathoinhibitory substance within the central nervous system (Patel et al., 2001). This is demonstrated by several lines of evidence.

NOS activity (in particular nNOS) has been demonstrated in central and peripheral sites involved in cardiovascular regulation throughout the autonomic nervous system by histochemical staining and immunohistochemistry (Bredt et al., 1990). Furthermore, several experimental studies examined the central effects of NO on sympathetic outflow by administration of NO agonists or blockers orally, intravenously, intracerebroventricularly, or into specific central sites (Patel et al., 2001; Hirooka et al., 2011). The vasoconstrictor and BP-raising effect of acute or chronic administration of exogenous NOS-inhibitors is well established (Huang et al., 1994). This effect could be at least in part due to vascular effects, namely impairment of NO basal vascular tone and endothelium-mediated vasodilation, but several experimental studies suggest that SNS activation may play a key role in hypertensive response to NO-blockade (Patel et al., 2001; Hirooka et al., 2011). Acute intravenous administration of NOS-inhibitors, such as L-NMMA, was found to induce BP increase and SNS activation, measured by means of serum norephinephrine assay and renal sympathetic nerve activity (SNA) (Sakuma et al., 1992). Both responses were further increased after baroceptor deafferentation and disappeared after cervical spine section, suggesting that the L-NMMA pressor effect is mostly due to its effect on SNS (Sakuma et al., 1992). This conclusion is supported by the evidence that ganglionic blockade (Cunha et al., 1993) and sympathectomy (Sander et al., 1995) suppressed BP and heart rate increase induced by L-NAME (another NOS-inhibitor). In contrast, few studies showed no changes in BP or SNS activity after pharmacological NOS-blockade (Liu et al., 1996).

Another confirmation of the putative dependency of hypertension induced by NOS-blockade on action of the central nervous system derives from experiments performed with intracerebroventricular administration of L-NAME. Thus, its hypertensive effect was blunted by β-blocker coadministration (Nurminen et al., 1997) and by cervical spine section (Togashi et al., 1992). Microinjection of NOS-inhibitors in the nucleus tractus solitarius (NTS), the origin of vagal efferent fibers, caused BP and renal SNA increase in rabbits undergoing or not undergoing barodenervation (Harada et al., 1993). NO modulation is also particularly relevant in rostroventrolateral medulla (RVLM), the main bulbar area of integration of excitatory autonomic efferent fibers involved in cardiovascular regulation. Thus, microinjection of NOS-inhibitors at this level caused renal SNA and BP increase, while NO-donors exhibited opposite effects (Zanzinger et al., 1995). Furthermore, hypothalamic paraventricular nuclei contain NOS-positive neurons, capable of modulating renal SNA (Zhang et al., 1997).

NO seems also to play a role in the pathophysiology of diseases characterized by increased sympathetic discharge. Experimental hypertension is associated with reduced activity of the intracerebral NO-pathway, as demonstrated in stroke-prone, spontaneously hypertensive rats, as well as in renovascular hypertensive rats (Hirooka et al., 2011). The same phenomenon occurs in experimental heart failure, which is characterized by decreased nNOS expression in the central nervous system (Zucker, 2006).

NO seems to exert its regulatory functions in the SNS even beyond the above-mentioned effects on central sympathetic outflow. NOS has been localized in sympathetic nerves, ganglia, and adrenal glands of Sprague-Dawley rats and pigs, suggesting that NO is released as a cotransmitter in the peripheral autonomic system (Dun et al., 1993; Modin et al., 1994). The key role of nitrergic innervations of vascular smooth muscle in neurogenic control of vascular function is reviewed in greater detail elsewhere (Toda and Okamura, 2003). Moreover, adrenergic receptors on endothelial cells, activated by circulating norepinephrine, can stimulate NO release (Miller and Vanhoutte, 1985). Finally, sympathetically-induced vasoconstriction may increase shear stress on the vascular wall, which in turn increases NO release from vascular endothelial cells. On the other hand, NO may play a role in the adrenergic system by enhancing the activity of norepinephrine neuronal reuptake in sympathetic nerve terminals (Simaan and Sabra, 2011).

### Reactive oxygen species

The half-life of NO, and therefore its biological activity, is critically influenced by the presence of ROS, such as superoxide. This free radical rapidly reacts with NO to form the highly reactive intermediate peroxynitrite (ONOO^−^). The formation of nitroso-compounds has multiple negative effects, reducing NO availability, exerting direct vasoconstrictor and cytotoxic effects, and impairing the activity of the prostacyclin synthase and eNOS (Munzel et al., 2010). Other ROS, such as the dismutation product of superoxide hydrogen peroxide and hypochlorous acid, cannot be considered as free radicals, but have a powerful oxidizing capacity, which further contributes to oxidative stress within vascular tissues (Munzel et al., 2010). The main sources of increased oxidative stress in cardiovascular diseases are the nicotinamide dinucleotide phosphate (NADPH) oxidase, the xanthine oxidase, mitochondria and, under certain conditions, even eNOS (Munzel et al., 2010).

Oxidative stress appears to stimulate central sympathetic outflow in various experimental models of hypertension, with little or no effect in control animals (Shokoji et al., 2003; Campese et al., 2005; Ye et al., 2006; Oliveira-Sales et al., 2008). Increased oxidative stress has been documented in specific nuclei of the brain involved in the regulation of sympathetic control of vasomotor tone in hypertensive but not normotensive rats (Kishi et al., 2004; Tai et al., 2005). Injection of antioxidants directly at this level results in a decrease in BP and SNA (Ye et al., 2006; Oliveira-Sales et al., 2008). Superoxide could act by reducing NO availability, in parallel in the central nervous system as well as in the vasculature (Zucker, 2006), although some studies also hypothesized an NO-independent effect (Xu et al., 2002).

Experimental studies have demonstrated that acute correction of oxidative stress, with vitamin C (Oliveira-Sales et al., 2008) or different antioxidants infused both intravenously and intracerebrally (Xu et al., 2002; Shokoji et al., 2003; Campese et al., 2005), can reduce SNS activity. Selective vitamin C injection in RVLM of renovascular rats reproduces all hemodynamic and sympatho-inhibitory effects of its systemic administration (Oliveira-Sales et al., 2008). Moreover other experimental studies suggest that the interaction between SNS and oxidative stress may occur not only in the brain stem, but also in the peripheral nervous system, such as preganglionic neurons (Lin et al., 2003), sympathetic ganglia (Cao et al., 2007), peripheral nerves (Shokoji et al., 2004), and neuroeffector junctions (Macarthur et al., 2008).

In recent decades the possible antihypertensive effect of antioxidants has been investigated in several studies, with conflicting results (Duffy et al., 1999; Fotherby et al., 2000; Kim et al., 2002; Ward et al., 2004; Hooper et al., 2008). This effect, attributed in the past mainly to vascular mechanisms such as restoration of NO-mediated vasodilation (Taddei et al., 1998; Hooper et al., 2008), could be at least in part a consequence of sympatho-inhibition achieved by antioxidants, as recently suggested also in humans (Bruno et al., 2012a).

### Renin-angiotensin system

Angiotensin-II is able to potentiate SNS activity at different levels (Grassi, 2001). In experimental studies intracerebral infusion of angiotensin-II caused hypertension associated with systemic vasoconstriction and a baroreflex reset towards higher BP levels (Reid, 1992). It was first demonstrated that vascular smooth muscle NADPH oxidase is activated by Angiotensin II and subsequently increases vascular ROS levels (Griendling et al., 1994). However, more recent work has shown that a similar phenomenon occurs within the central nervous system, where Angiotensin-II up-regulates NADPH oxidase via activation of type 1 (AT1) receptors (Zucker, 2006). Importantly, this process has been shown to occur in neuroanatomical areas implicated in central sympathetic regulation such as the RVLM, the circumventricular organs, and the paraventricular nuclei (Gao et al., 2005; Li et al., 2006; Zucker, 2006). Zucker and colleagues have suggested that this pathway becomes very important in heart failure and contributes to the well-known reductions in arterial baroreflex sensitivity and increases in central sympathetic drive present in this disease state (Gao et al., 2005; Zucker, 2006). Overall, these animal investigations indicate that increases in central sympathetic outflow in heart failure are mediated via Angiotensin II activation of NADPH oxidase and subsequent production of ROS, which may directly activate central SNS pathways along with scavenging NO, thereby removing the tonic restraint on sympathetic outflow (Fisher et al., 2009). Emerging evidence indicates that both AT1 and AT2 receptors are involved in sympathetic discharge regulation in the RVLM during heart failure (Gao et al., 2005, 2008).

Furthermore, at the peripheral level Angiotensin-II facilitates neuronal transmission within sympathetic ganglia (Reit, 1972; Reid, 1992), favors norepinephrine release by sympathetic nerve terminals, acting on pre-synaptic receptors (Starke, 1977) and enhances α-mediated vasoconstriction in arterioles (Grassi, 2001). The latter phenomenon was also demonstrated in humans (Taddei et al., 1995; Saino et al., 1997).

### Endothelin

Endothelin-1 (ET-1) is a vasoconstrictor and mitogenic peptide produced by endothelial cells and its important role in regulation of vascular tone and structure is well established (Dhaun et al., 2008). Essential hypertension is characterized by increased ET-1 vasoconstrictor tone (Cardillo et al., 1999; Taddei et al., 1999), which seems to be a consequence of reduced NO availability (Taddei et al., 1999). The role of the ET system in cardiovascular homeostasis is not limited to its direct vascular effects, but also involves the neural regulation of vasomotor tone (Mosqueda-Garcia et al., 1993). Experimental evidence suggests that ET-1 can stimulate central and peripheral SNS activity through ET_A_ receptors (Gulati et al., 1997; Nakamura et al., 1999). While intracerebral administration of ET-1 can increase BP and SNS activity mainly through ET_A_ receptors in hypertensive as well in normotensive animals (Gulati et al., 1997; Nakamura et al., 1999), the administration of an ET_A_ receptor antagonist determines the opposite effect only in hypertensive animals, suggesting a specific sympathoexcitatory role for the endogenous ET system in this condition (Nakamura et al., 1999). With regard to the peripheral autonomic nervous system, ET-1 can act in carotid bodies and in cervical superior and nodose ganglia, influencing baroreflex and chemoreflex regulation. ET-1 is also released by post-ganglionic sympathetic neurons, possibly modulating catecholamine release and vascular tone, and stimulates catecholamine release from adrenal glands (Mortensen, 1999).

## Endothelial function and sympathetic nervous system activity in humans: a cause-effect relationship?

Reduced availability of NO in the vasculature is present in several cardiovascular risk factors and diseases and leads to endothelial dysfunction, which is the first step of the atherosclerotic process. Endothelial dysfunction is increasingly accepted as a common trait of essentially all cardiovascular risk factors. Impaired endothelial homeostasis (mostly demonstrated under the form of abnormal vasomotor responses) has been shown, among other individuals, in the elderly, after chronic or acute smoking, in patients with hypercholesterolemia or hypertriglyceridemia, in patients with Type I and II diabetes mellitus, hypertension and metabolic syndrome (Brunner et al., 2005) (Table 1). A number of different techniques have been developed to assess endothelial function in humans, including biochemical markers, genetic markers, vascular reactivity tests: these methodological aspects are reviewed in greater detail elsewhere (Deanfield et al., 2005). This section will review the main advances in elucidating the relationship between endothelial function and SNS activity in humans, obtained by microneurographic recordings, allowing direct quantification of MSNA.

**Table 1:** **Conditions/diseases characterized by both autonomic and endothelial dysfunction and interventions known to ameliorate both autonomic and endothelial dysfunction**.

**Conditions/diseases characterized by both autonomic and endothelial dysfunction**	**Interventions known to ameliorate both autonomic and endothelial dysfunction**
Aging Menopause Hypertension Obesity Diabetes Metabolic syndrome Smoking Obstructive sleep apnea syndrome Ischemic heart disease Heart failure Chronic kidney disease Pre-eclampsia Chronic obstructive pulmonary disease Pulmonary artery hypertension Hepatic cirrhosis Hypothyroidism	Physical exercise Weight loss Estrogen replacement therapy Renin-angiotensin system blockers Endothelin antagonists Antioxidants Statins Sibutramine Central sympatholytic drugs

### Evidence in healthy humans

There is indirect evidence of a reciprocal relationship between endothelial function and the activity of the SNS. Men tend to have higher sympathetic outflow than women (Ng et al., 1993; Narkiewicz et al., 2005), who in turn have higher endothelial function, evaluated in the small resistance arteries as well as in conduit arteries (Virdis and Taddei, 2012). Furthermore, vascular aging and sympathetic traffic show similar behavior over time in males and females (Ng et al., 1993; Narkiewicz et al., 2005; Virdis and Taddei, 2012). Sympathetic nerve activity is augmented in the early morning before waking, while endothelial function has been reported to be attenuated (Somers et al., 1993; Otto et al., 2004).

Several studies explored the effects of basal NO release on MSNA. Intravenous administration of NOS inhibitors in healthy humans initially appeared to have no effect on SNS activity, evaluated by means of microneurography (Hansen et al., 1994), while further studies suggested a sympathoexcitatory effect (Owlya et al., 1997). This apparent contradiction was then explained by the demonstration that different L-NMMA dosages exerted different effects on humans. Lepori and coauthors (Lepori et al., 1998) administered intravenously increasing L-NMMA dosages, and equipressor phenylephrine dosages, in healthy volunteers. High L-NMMA doses suppressed MSNA to a similar extent as phenylephrine. In contrast, low L-NMMA doses induced no changes in MSNA, even in the presence of a significant BP increase. When a vasodilator, such as sodium nitroprusside, was co-infused with L-NMMA in order to avoid any BP modifications, an increase in MSNA was evident (Owlya et al., 1997). Taken together, these data suggest that L-NMMA infusion at high doses causes mainly direct peripheral vasoconstriction, while low doses were able to reveal an excitatory effect on SNS, demonstrating that in physiological conditions NO can modulate vascular tone both at the neuronal and endothelial level. In particular, NO seems to act in the central nervous system by influencing tonic sympathetic discharge, without influencing the efficacy of reflex responses. Thus, during intravenous L-NMMA infusion, MSNA changes during pharmacological baroreceptor activation and deactivation (Miyano et al., 1997), as well as during tilt test maneuvers (Cui et al., 2003), lower-body negative pressure (Spieker et al., 2000), and handgrip test (Owlya et al., 1997), were preserved. In contrast, L-NMMA infusion blunted hypertensive and sympathoexcitatory responses to mental stress, through unknown mechanisms (Lindqvist et al., 2004). Baroreceptor regulation of heart rate during lower-body negative pressure was also altered after inhibition of NO synthesis in healthy volunteers, suggesting an important role of NO in heart rate regulation in humans (Spieker et al., 2000). Interestingly, altered baroreflex regulation of heart rate, but not of MSNA, is a typical feature of endothelial dysfunction-related states, such as arterial hypertension (Grassi et al., 1998), a condition mimicked by NOS-inhibition.

Interaction between NO and SNS in humans seem to occur not only in the central nervous system, but also at the peripheral level. In subjects undergoing thoracic sympathectomy for hyperhidrosis, vasoconstriction to intravenous L-NMMA administration is potentiated (Lepori et al., 1998), suggesting that sympathetic innervation is physiologically able to inhibit vasoconstriction induced by NOS-inhibition.

Some authors suggested that interaction between NO and SNS could explain, at least in part, large interindividual differences in resting MSNA among normotensive humans with similar BP, thus elucidating the role of SNS in long-term control of BP (Skarphedinsson et al., 1997; Charkoudian et al., 2006; Joyner et al., 2008). Skarphedinsson and coauthors found a significant positive correlation between plasma nitrates and resting MSNA in 22 healthy young volunteers. This led to the hypothesis that greater basal NO vascular tone could counteract excessive vasoconstriction induced by adrenergic overactivity, resulting in similar BP values with different sympathetic discharge levels (Skarphedinsson et al., 1997). Charkoudian and coauthors recently implemented this hypothesis, introducing cardiac output as a cofactor. In this study L-NMMA was infused intravenously in 18 healthy normotensive volunteers with resting MSNA varying from 13 to 68 bursts/100 heartbeats. In subjects with high resting MSNA, L-NMMA administration induced an almost twofold increase in BP as compared to those with low MSNA (Charkoudian et al., 2006). This effect was not associated with similar increase in total peripheral resistances, indicating similar vasoconstriction, but with different cardiac output. In particular, subjects with high MSNA had lower resting cardiac output, and a lower decrease in cardiac output during L-NMMA administration (Charkoudian et al., 2006). Moreover cardiac output, which is a key determinant of resting BP together with sympathetic tone (Charkoudian et al., 2006), is also NO-dependent, since it was decreased after NOS-inhibition (Spieker et al., 2000). Thus, it is conceivable that in pathological conditions in which NO basal vascular tone is altered, subjects with higher baseline MSNA could be at greater risk of developing hypertension.

**Figure 2:**
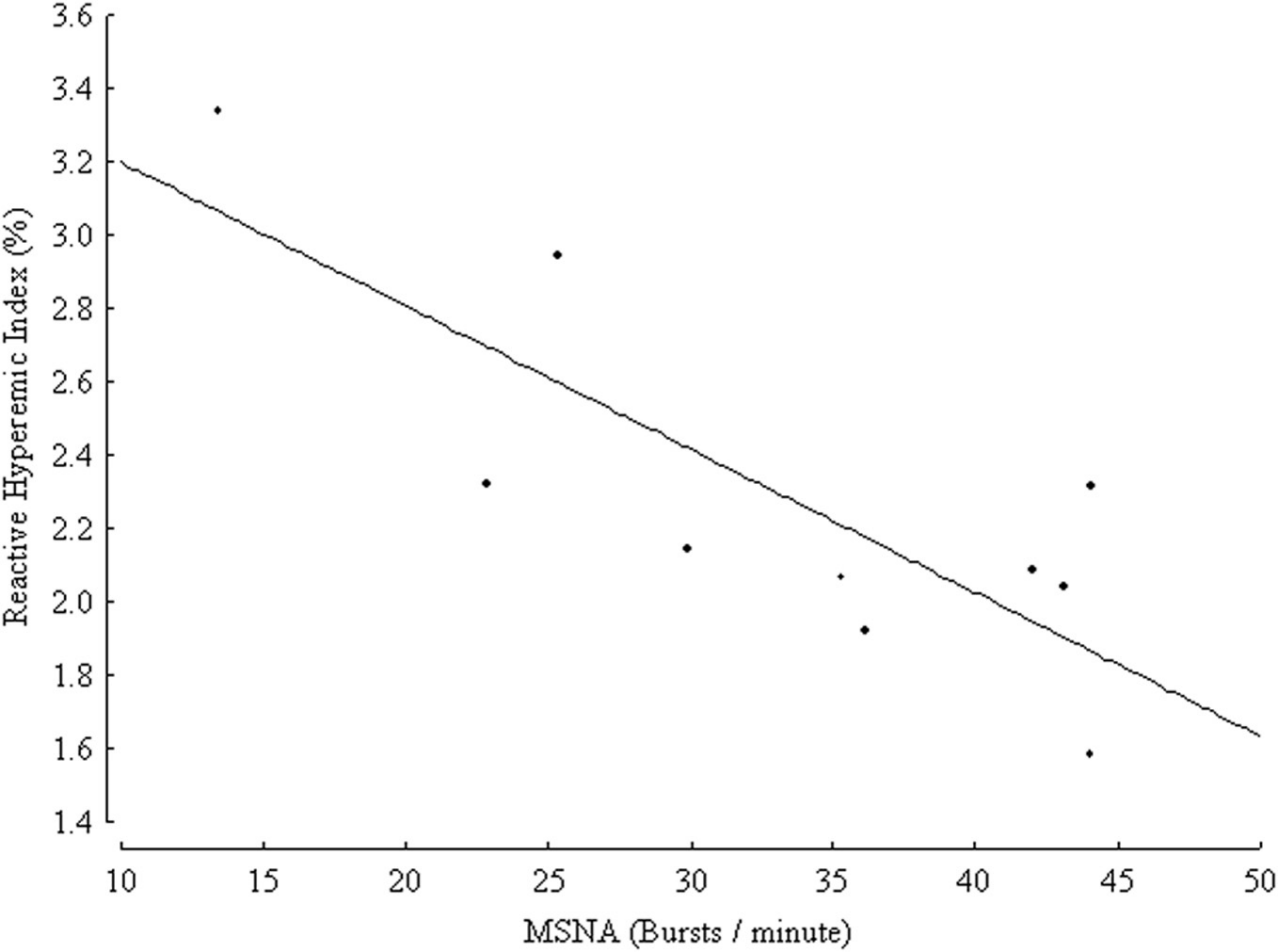
**Inverse relationship between muscle sympathetic nerve activity (MSNA) expressed as burst frequency (bursts/min) and reactive hyperemic index (%) in 10 healthy controls, (*r* = 0.8, *p* = 0.005).** (From Sverrisdottir et al., 2010, CC-BY license).

Very recently, an inverse relationship between markers of endothelial function and of sympathetic activity in healthy conditions has been suggested. In a group of 314 healthy subjects endothelial function in the brachial artery was inversely related to plasma norepinephrine concentration (Kaplon et al., 2011). The above-mentioned correlation was significant in women but not in men even after controlling for age, common cardiovascular risk factors and oxidative stress, suggesting that sympathetic activity could be a gender-specific determinant of vascular function (Kaplon et al., 2011). In the cited study, conduit artery endothelial function was measured by means of a non-invasive brachial artery reactivity test known as flow-mediated dilation (FMD), in which NO release is stimulated by post-ischemic reactive hyperemia, and the measured endpoint is constituted by the percent change in brachial artery diameter, obtained by ultrasound (Deanfield et al., 2005; Thijssen et al., 2011). FMD is one of the most widely used techniques of endothelial function assessment, and a recent meta-analysis demonstrated that it is an independent predictor of cardiovascular events in the general population as well as in subjects with cardiovascular risk factors or disease (Inaba et al., 2010). The correlation between markers of endothelial function and SNS activity was also demonstrated in one microneurographic study (Sverrisdottir et al., 2010). In a group of ten healthy volunteers, with age ranging from 24 to 61 years, MSNA was inversely related to reactive hyperemia index, obtained by finger photoplethysmography (Sverrisdottir et al., 2010) (Figure 2). The device records pulse wave amplitude at baseline and during reactive hyperemia following arm arterial occlusion. The reactive hyperemia index is a non-invasive index of microvascular endothelial function. Its association with classical cardiovascular risk factors was validated in large cohorts but its prognostic role is yet to be established (Reriani et al., 2010). The relationship between MSNA and reactive hyperemia index was independent of age and sex, but related to habitual physical activity (Sverrisdottir et al., 2010).

Physical activity has been demonstrated to be protective against the development of cardiovascular events. The mechanisms involved include reduction of sympathetic outflow and restoration of endothelial function (Cornelissen and Fagard, 2005; Mora et al., 2007). A metaanalysis of clinical trials reported that the effects of exercise training on BP, driven by peripheral vascular resistance reduction, are accompanied by reduction in heart rate and norephinephrine levels (Cornelissen and Fagard, 2005). These data based on non-invasive and indirect parameters were confirmed in smaller studies using direct neural recordings by microneurography. In hypertensive patients, a program of exercise training consisting of three 60 min exercise sessions per week for 4 months reduced BP and MSNA and restored baroreflex sensitivity (Laterza et al., 2007). Exercise training is known to ameliorate endothelial dysfunction in healthy subjects (Clarkson et al., 1999), as well as in the presence of well known risk factors (Higashi et al., 1999; Taddei et al., 2000; Franzoni et al., 2005) and established cardiovascular diseases (Hornig et al., 1996; Hambrecht et al., 2003). To date, it is not clear whether physical activity acts by inducing sympatho-inhibition and restoration of vascular function in parallel, or rather influences the one by means of the other. This important aspect needs to be elucidated in further studies.

### Endothelial function during sympatho-excitatory maneuvers

One-Way to investigate the relationship between sympathetic vasoconstrictor activity and NO formation is to measure markers of endothelial function during maneuvers known to increase sympathetic nerve traffic. However, this indirect approach has achieved conflicting results. In 16 young, healthy volunteers, brachial artery FMD was measured in resting conditions and during lower-body negative pressure (Hijmering et al., 2002). FMD, but not vasodilation to nitrates (which is endothelium-independent), was markedly reduced by this baroreceptor-unloading maneuver. Furthermore, vascular response during lower-body negative pressure was blunted by phentolamine infusion, which in turn had no effect on resting FMD (Hijmering et al., 2002). In contrast, in young healthy volunteers, FMD in the femoral artery was not modified by sympathetic activation, obtained by cold pressor test, a potent non-baroreflex sympathoexcitatory stimulus (Victor et al., 1987), or deactivation, obtained after maximal cycling exercise, whereas this did occur in older healthy subjects (Thijssen et al., 2006). Mental stress is a powerful stimulus for MSNA increase (Anderson et al., 1987; Hjemdahl et al., 1989), possibly as a result of a primary central sympathetic excitation (Wallin et al., 1992). Acute mental stress also induces transient but sustained endothelial dysfunction, lasting up to 4 h, accompanied by BP, heart rate, and salivary cortisol increase (Ghiadoni et al., 2000). This long-lasting effect was prevented by selective endothelin-A receptor antagonism (Spieker et al., 2002), which was demonstrated to achieve sympatho-inhibition (Bruno et al., 2011).

A possible mechanism explaining discrepancies in endothelial function behavior during different sympatho-excitatory maneuvers was suggested in a recent study (Padilla et al., 2010). Fourteen young healthy men performed three sympatho-excitatory maneuvers: graded lower body negative pressure, cold pressor test, and 35% maximal voluntary contraction handgrip followed by post-exercise ischemia. Lower body negative pressure, a neutral maneuver as far as BP is concerned, induced a proportional increase in MSNA and oscillatory/retrograde shear stress. During the cold pressor test and handgrip, on the other hand, the concomitant hypertensive effect probably masked this phenomenon, which is able to cause endothelial dysfunction in conduit arteries (Padilla et al., 2010). Thus, further microneurographic studies, allowing continuous recordings during acute challenges and simultaneous vascular acquisitions, could help to elucidate the acute effect of SNS activation on vascular function, clarifying apparently contrasting findings. A more detailed study of physiological behavior during acute sympathoexcitatory maneuvers could suggest pathways involved in chronic autonomic disregulation in cardiovascular diseases.

### Evidence in diseases characterized both by endothelial dysfunction and increased sympathetic nerve traffic

Another appropriate way to investigate the relationship between vasoconstrictor nerve activity and NO formation would be to study states of chronically increased sympathetic nerve traffic. Virtually all cardiovascular risk factors and diseases in which increased adrenergic drive was demonstrated, including hypertension (Grassi et al., 1998), obesity (Grassi et al., 2004), chronic kidney disease (Ligtenberg et al., 1999), heart failure (Grassi et al., 1995), are also characterized by endothelial dysfunction (Brunner et al., 2005), as well as many other cardiovascular and non-cardiovascular diseases (Table 1), Furthermore, several pharmacological and non-pharmacological interventions are known to ameliorate both autonomic and vascular dysfunction (Table 1). Up to now, few studies have directly investigated possible interrelationships between SNS and vascular function in disease conditions.

In essential hypertension, increased SNS activity is one of the main mechanisms responsible for the pathogenesis of the disease and the development of target organ damage (Grassi et al., 1998; Grassi, 2009). Studies performed by using direct as well as indirect approaches to assess neuroadrenergic function have almost univocally shown that the sympathetic overdrive is detectable not only in borderline but also in mild to moderate and in more severe essential hypertensive patients, varyingin parallel with the magnitude of the BP increase. Increased sympathetic discharge is peculiar to the essential hypertensive state and involves each district studied (muscle, brain, kidney, heart), except the skin district (Grassi et al., 1998; Grassi, 2009). Although several studies support the hypothesis that increased sympathetic discharge originates mainly in the central nervous system (Ferrier et al., 1992), it is also true that human hypertension is characterized at the peripheral level by down-regulation of peripheral α-adrenergic receptors, impairment in the neuronal reuptake of norepinephrine from sympathetic nerve terminals, and altered functional interaction at the level of the vascular wall between norepinephrine, epinephrine, and other humoral (such as angiotensin II), metabolic (including insulin and leptin), or endothelium-derived substances (Grassi, 2009). However, the mechanisms underlying such alterations are not yet fully understood. Another pathophysiological characteristic of essential hypertension is the presence of endothelial dysfunction, demonstrated at the coronary and peripheral level, in the micro- and macrocirculation (Versari et al., 2009). Reduced NO availability, which is at the basis of endothelial dysfunction, is mainly caused by increased vascular oxidative stress (Taddei et al., 1998), but ET-1 and other endothelium-derived contracting factors are also involved (Versari et al., 2009).

Up to now, only few preliminary studies have investigated the NO-SNS relationship in hypertensive patients. Gamboa and colleagues set up an elegant experimental protocol (evaluation of BP responses to L-NMMA infusion during ganglionic blockade) in order to ascertain the contribution of basal NO tone in BP regulation independently of interaction with SNS, in physiological and pathological states in humans. They found that NO tonically restrains BP by about 30 mmHg, with no differences between normotensive, pre-hypertensive and hypertensive patients (Gamboa et al., 2012). The authors concluded that if NO deficiency contributes to hypertension, it is likely to be due to interactions with the autonomic nervous system, which were excluded in the study (Gamboa et al., 2012). However, this indirect suggestion needs to be confirmed in other studies.

**Figure 3:**
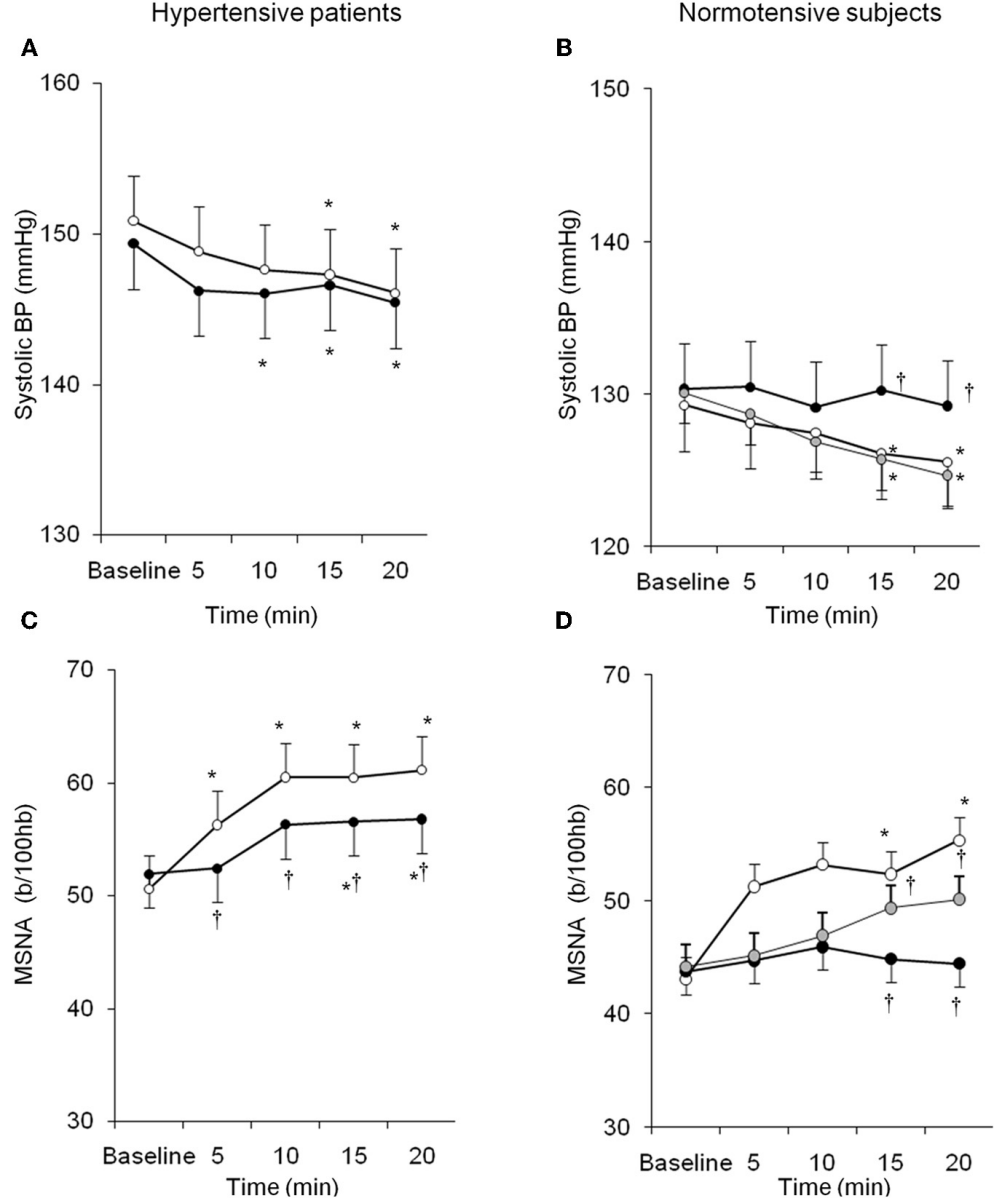
**Behavior of systolic BP and MSNA in hypertensive patients and normotensive subjects during BQ123 (an endothelin-A receptor antagonist) infusion at 0.1 mg/kg per hour (black circles) and 0.2 mg/kg per hour (gray circles), as well as during sodium nitroprusside (white circles) infusion at equidepressor doses.** Data are shown as mean ± SEM. b/100 hb: bursts per 100 hb; ^*^P0.05 vs. baseline; ^†^*P*0.05 vs. sodium nitroprusside. (From Bruno et al., 2011, with permission).

Similarly, despite the growing body of evidence from experimental studies, few data are available on the systemic interaction between endogenous ET-1 and SNS in humans either in physiological or pathological conditions. Interestingly, local infusion of ET-1 is able to potentiate SNS-mediated vasoconstriction induced by deep breath (Haynes et al., 1994). We recently demonstrated that ET-1 likewise modulates sympathetic activity in humans through ET_A_ receptors, and that this interaction is peculiar to the hypertensive status (Bruno et al., 2011). In 15 untreated patients with essential hypertension and in 10 healthy subjects MSNA was recorded in resting conditions and during acute intravenous infusion of BQ123, an ET_A_–receptor antagonist. BQ123 induced BP decrease in hypertensive and normotensive subjects, accompanied by a blunted increase in SNS activity (Figure 3). This finding suggests that endogenous ET-1, by the stimulation of ET_A_ receptors, contributes to the basal sympathetic tone that controls vascular resistances in humans. Moreover in hypertensive subjects as compared to the control group, lower doses of BQ123 were sufficient to reveal the vasodilating and sympatho-inhibitory effect of ET_A_ blockade. Suggesting that essential hypertension is characterized by a greater susceptibility to the sympatho-excitatory effect of endogenous ET-1 through activation of the ET_A_ receptor subtype (Bruno et al., 2011). Taken together, these findings suggest that in the presence of essential hypertension, the increased biological activity of endogenous ET-1 takes place in parallel in different systems, such as in the peripheral vasculature and in the SNS.

Very recently interactions between the oxidative stress system and SNS were investigated in a microneurographic study enrolling 32 essential hypertensive patients and 22 healthy volunteers (Bruno et al., 2012a). In this study high dose Vitamin C infusion significantly lowered BP and MSNA in hypertensive patients but not in normotensive subjects. Sympatho-vagal balance and spontaneous baroreflex sensitivity were restored during vitamin C infusion in hypertensive subjects but not in healthy subjects. These results demonstrated that acute administration of vitamin C is able to reduce cardiovascular adrenergic drive in hypertensive patients, suggesting that oxidative stress is involved in the regulation of sympathetic activity in essential hypertension. In contrast, this pathophysiological mechanism is not present in healthy conditions (Bruno et al., 2012a), as also suggested by previous studies (Bell et al., 2003).

**Figure 4:**
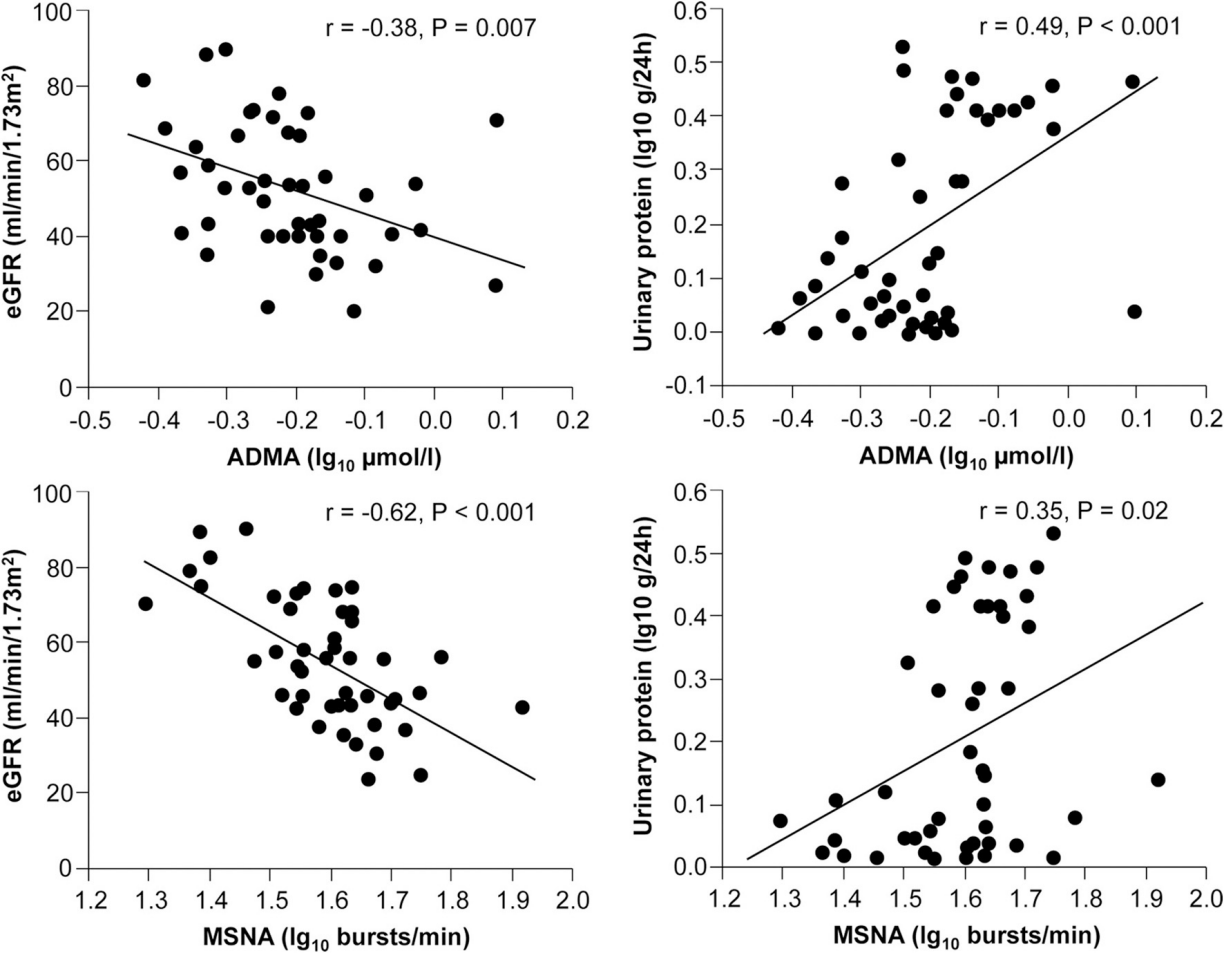
**Relationships between muscle sympathetic nerve activity (MSNA), asymmetric dimethylarginine (ADMA), and estimated GFR (eGFR) or proteinuria in 48 stage 2–4 CKD patients (From Grassi et al., 2011b, with permission)**.

Sympathetic activation is a feature of chronic kidney disease both in early and advanced stages (Converse et al., 1992; Grassi et al., 2011a), representing one of the most powerful predictors of mortality and cardiovascular events (Zoccali et al., 2002). Increased neuroadrenergic drive is independent of circulating uremia related toxins and probably related to afferent nerve signals from ischemic kidneys, decrease in NO bioavailability, stimulation of carotid chemoreceptors by metabolic acidosis and activation of the renin-angiotensin system (Kotanko, 2006). The endothelium-SNS relationship was explored in 48 patients with chronic kidney disease of different etiologies (Grassi et al., 2011b). In this population, the tertile with highest sympathetic nerve traffic values also showed the highest ADMA levels, and this association was paralleled by a continuous, positive relationship between these two parameters, independently of other confounders. Both sympathetic nerve traffic and ADMA were inversely related to the estimated glomerular filtration rate and directly related to left ventricular geometry (Figure 4).

Remarkably, in a multiple regression model including both variables, variance of the estimated glomerular filtration rate, proteinuria, and left ventricular geometry explained by sympathetic nerve traffic and ADMA largely overlapped, because sympathetic nerve traffic but not ADMA was retained as significant (Grassi et al., 2011b). These results suggest that in chronic kidney disease the relationship between sympathetic activity and vascular function can be of crucial importance for progression of renal damage. This hypothesis is confirmed in a prospective study involving 224 hemodialysis patients followed up for about 3.5 years (Mallamaci et al., 2004). In this cohort plasma norepinephrine and ADMA were both predictors of death and cardiovascular events, and their prognostic significance largely overlapped (Mallamaci et al., 2004). Moreover, systemic intravenous infusion of ADMA increased renal SNA in rats (Augustyniak et al., 2006), suggesting that in conditions in which ADMA concentrations are elevated, NO-mediated regulation of central sympathetic outflow could be impaired. Recent observations showed that the renal function decline over time in non-proteinuric nephropathy-like hypertensive nephrosclerosis was accelerated by adrenergic genetic influences (Chen et al., 2010), and that angiotensin-converting enzyme inhibition, an intervention that markedly inhibits the SNS in CKD patients (Ligtenberg et al., 1999), determined a parallel decline in proteinuria and ADMA levels in patients with diabetic nephropathy and preserved glomerular filtration rate (Yilmaz et al., 2010).

There is some evidence that anxiety disorders are associated with an increased cardiovascular risk (Fleet et al., 2000) and the pathophysiology could be linked to autonomic alterations. Whole-body and regional sympathetic nervous activity are not elevated at rest, nor during mental stress, in patients with panic disorder. On the other hand, epinephrine spillover from the heart at rest is increased, while quantification from single vasoconstrictor unit recording provided evidence of a disturbed resting sympathetic firing pattern in patients with panic disorder (Lambert et al., 2006). Moreover, patients with panic disorder exhibited a loss in heart rate variability (Gorman and Sloan, 2000) and an enhanced reflex gain of the arterial baroreflex control of MSNA but no change in cardiac baroreflex sensitivity (Lambert et al., 2002). Anxiety was also shown to be associated with endothelial dysfunction via autonomic dysregulation, evaluated by spectral analysis of heart rate variability in 41 subjects (Narita et al., 2007). A recent study investigated the single-fiber and multi-fiber pattern of muscle sympathetic firing in 8 women and 17 men with metabolic syndrome and high BP (Lambert et al., 2010). Women had higher cholesterol levels, higher depressive symptom scores and similar multiunit MSNA compared with men, but displayed a disturbed firing pattern of sympathetic activity as indicated by a higher incidence of multiple spikes per burst. Multiple firing during a sympathetic neural burst was associated with higher trait anxiety score and higher affective depressive symptoms (Lambert et al., 2010). Since gender plays a crucial role in the association between metabolic syndrome and vascular dysfunction (Plantinga et al., 2008), these data reinforce the role of gender-specific interaction between vascular function and SNS in explaining the link between anxiety and cardiovascular risk.

In conclusion, several diseases are characterized by both endothelial dysfunction and sympathetic traffic (Table 1). However, a direct relationship between these two alterations has so far been demonstrated in few conditions. Future research should investigate this aspect in other diseases, such as obesity, obstructive sleep apnea, heart failure, and many others. In particular, future studies should clarify the presence of a cause-effect relationship, or of common causative mechanisms. Involvement of different pathways underlying sympathetic/vascular activation in different diseases may lead to specific treatment strategies.

## Arterial stiffness and sympathetic nerve traffic

The two main functions of the arteries, namely the conduit function (to deliver an adequate supply of blood to peripheral tissues) and the cushioning function (to buffer pressure oscillations due to intermittent ventricular ejection), are closely related to arterial elastic properties, which permit the generation and propagation of a BP wave along the arterial walls (Nichols and O'Rourke, 2005). At the level of bifurcations and areas of turbulence, the pulse wave is reflected and retrograde waves are generated, which, summed with the forward wave generated by ventricular ejection, constitute the effective BP curve, contributing to the amplitude of pulse pressure and systolic BP (Nichols and O'Rourke, 2005). The velocity of pulse wave propagation is inversely related to large artery distensibility; this variable, the so-called pulse wave velocity (PWV), as well as timing and magnitude of wave reflection (expressed as the augmentation index) can now be measured easily and non-invasively, becoming probably the most widely used technique for assessment of vascular function and structure (Laurent et al., 2006). The ageing process is characterized by arteriosclerosis, a profound remodeling of arterial walls, associated with structural changes such as increased collagen deposition and rupture of elastin fibers in the vascular wall (Safar et al., 2003; Nichols and O'Rourke, 2005). Systemic arterial stiffness reflects the overall opposition of large arteries to the pulsatile effects of ventricular ejection. The consequences are increased left ventricular afterload with left ventricular hypertrophy, reduced coronary perfusion with aggravation of ischemia and progressive atherosclerosis (Ghiadoni et al., 2009). As well as aging, several chronic disease states such as hypertension diabetes, hypercholesterolemia, obesity, metabolic syndrome, are associated with increased arterial stiffness (Laurent et al., 2006). Both aortic PWV and the augmentation index proved to be independent predictors of cardiovascular events in a recent metaanalysis (Vlachopoulos et al., 2010).

In addition to structural changes, arterial stiffness is strongly affected by vascular smooth muscle cell tone, which is in turn regulated both by endothelial cell signaling and the SNS (Wilkinson and McEniery, 2004). Sympathetic stimulation may influence arterial wall mechanics both indirectly, by passively increasing arterial pressure, or directly, by changing smooth muscle cell tone (Boutouyrie et al., 1994; Joannides et al., 1995; Lydakis et al., 2008). Boutouyrie and coauthors demonstrated that radial artery diameter decreased during sympathoexcitatory maneuvers such as cold pressor and mental stress tests (Boutouyrie et al., 1994), while other studies found a decrease in radial artery stiffness during the cold pressor test (Joannides et al., 1995). Conversely, in 13 young healthy subjects, wave reflection parameters exhibited different behavior during static exercise or lower body negative pressure, with timing of reflected wave and PP amplification being increased during the former and unchanged during the latter (Lydakis et al., 2008). The authors concluded that central arterial wall hemodynamics is linked to BP changes rather than sympathetic tone *per se* (Lydakis et al., 2008).

On the other hand, acute withdrawal of sympathetic tone caused increased large artery elasticity, as demonstrated in sympathectomized rats (Mangoni et al., 1997), but also in healthy and atherosclerotic subjects during brachial plexus or subarachnoid anaesthesia or one month after removal of the lumbar sympathectomy chain (Failla et al., 1999). Endothelium-derived factors such as NO (Wilkinson et al., 2002) and ET-1 (McEniery et al., 2003) have been proposed as physiological modulators of arterial stiffness in healthy individuals. An inverse correlation between endothelial dysfunction and arterial stiffness has been reported in cross-sectional studies performed in healthy subjects, with conflicting results (McEniery et al., 2006; Koivistoinen et al., 2012), as well as in patients with cardiovascular risk factors such as diabetes (Bruno et al., 2012b). In contrast, in healthy volunteers, the reduction in brachial artery distensibility elicited by lower body negative pressure and a cold pressor test was not influenced by administration of exogenous NO-donors (Salzer et al., 2008).

Taken together, these studies suggest that the relationship between arterial stiffness and sympathetic nerve traffic could be mediated by endothelium-related mechanisms. However, the evidence in this field is far from being conclusive and mechanistic studies are still required.

In 16 healthy subjects, neither brachial artery distensibility nor carotid artery distensibility were related to MSNA (Kosch et al., 2002). Conversely, in 25 healthy male subjects multiple linear regression analysis revealed that MSNA was an independent determinant of carotid to femoral PWV (Swierblewska et al., 2010). Given the paucity of data on this topic, it is not possible to ascertain reasons for these discrepant results. However, the different districts examined in the two studies, as well as gender differences in the study population, may be important factors to take into account. The latter hypothesis is reinforced by a recent study by Casey and coauthors (Casey et al., 2011). This study shows for the first time a gender-specific relationship between augmentation index and sympathetic vascular tone in healthy humans. This finding is of particular interest since it provides an explanation for better cardiovascular outcomes in women, who appear to be protected from negative effects of sympathetic activation on arterial hemodynamics. The authors recruited 44 young healthy subjects who underwent microneurography and applanation tonometry to obtain wave reflection parameters. MSNA was directly related to the augmentation index and total peripheral resistances in men while, interestingly, the relation between MSNA and augmentation index was inverse in women. Thus, the authors hypothesized that MSNA is able to compromise wave reflection, which is a negative prognostic factor for cardiovascular disease, only in men (Casey et al., 2011). However, further physiopathological studies are required to explore the mechanisms responsible for this gender-specific phenomenon.

Not only acute perturbations of SNS activity, as described above, can influence vascular stiffness, but chronic alterations of sympathetic discharge may also have a trophic effect on the vascular wall, increasing arterial stiffness (Bruijns et al., 1998). Consistent with the latter hypothesis, some animal studies showed a reduction in arterial distensibility in long-term sympathectomized rats (Lacolley et al., 1995). This hypothesis could additionally be tested in diseases characterized by chronically increased sympathetic traffic. However, to our knowledge, only one study investigating the relationship between arterial stiffness and adrenergic drive in this setting has been published (Kosch et al., 2002). In renal transplant recipients, who showed both a major impairment of large artery elastic wall properties and sympathetic overactivity, a relationship was found between sympathetic activity, measured by microneurography, and large artery distensibility, in muscular arteries, such as the brachial artery, but not in elastic arteries such as the carotid artery (Kosch et al., 2002). The correlation between brachial artery distensibility and MSNA remained statistically significant independently of arterial diameter, BP, graft function, sex, body mass index and smoking habits (Kosch et al., 2002). However, aortic stiffness in end stage renal disease displays a different pathophysiology and anatomical substrate as compared to other morbid conditions. Thus, the main muscular arterial involvement in the relationship with MSNA is not surprising.

Finally, is it important to notice that the relation between arterial stiffness and sympathetic activity appears to be bidirectional. Large artery stiffness can interfere with autonomic regulation by impairing carotid baroreflex sensitivity (Chapleau et al., 1995). This hypothesis was demonstrated for cardiovagal baroreflex sensitivity (Mattace-Raso et al., 2007) and recently confirmed also for sympathetic baroreflex sensitivity in a study using microneurography for quantification of adrenergic drive and ultrasound and magnetic resonance for evaluation of carotid and aortic distensibility in 61 elderly subjects (Okada et al., 2012).

## Conclusions

A crucial role of SNS in regulation of vascular function, in addition to the reflex regulation of vasomotor tone, is suggested by several lines of evidence. First, the same pathways are involved in central and peripheral autonomic regulation as well as in vascular function regulation: NO, ROS, ET, and the renin-angiotensin system. Second, markers of vascular dysfunction are inversely related to quantification of sympathetic discharge. Third, sympatho-excitatory maneuvers impair endothelial function. Fourth, several cardiovascular diseases are characterized by vascular dysfunction as well as sympathetic overactivity. However, there is still a paucity of data in humans on common regulating pathways. Furthermore, so far the studies demonstrating an association between vascular dysfunction and sympathetic activation evaluated mainly surrogate markers and enrolled very small cohorts. The physiopathology of sympathetic regulation of vascular function in cardiovascular diseases, almost completely unexplored at the moment, appears to be an intriguing field of research, with possible implications for treatment of these conditions.

In conclusion, although microneurographic recordings have allowed great advances in knowledge of mechanisms involved in sympathetic regulation of vascular function, this field is still largely unexplored in humans, both in physiological and pathological conditions.

### Conflict of interest statement

The authors declare that the research was conducted in the absence of any commercial or financial relationships that could be construed as a potential conflict of interest.
